# Hydrogen Stabilization and Activation of Dry-Quenched Coke for High-Rate-Performance Lithium-Ion Batteries

**DOI:** 10.3390/nano12193530

**Published:** 2022-10-09

**Authors:** Decai Qin, Fei Huang, Guoyin Zhu, Lei Wang

**Affiliations:** 1 School of Chemistry and Materials Science, Nanjing Normal University, Nanjing 210023, China; 2 Institute of Advanced Materials and Flexible Electronics (IAMFE), School of Chemistry and Materials Science, Nanjing University of Information Science & Technology, Nanjing 210044, China

**Keywords:** dry-quenched coke, hydrogen, active defects, stabilization, lithium-ion batteries

## Abstract

Lithium-ion batteries (LIBs) have rapidly come to dominate the market owing to their high power and energy densities. However, several factors have considerably limited their widespread commercial application, including high cost, poor high-rate performance, and complex synthetic conditions. Herein, we use earth-abundant and low-cost dry-quenched coke (DQC) to prepare low-crystalline carbon as anode material for LIBs and tailor the carbon skeleton via a facile green and sustainable hydrogen treatment. In particular, DQC is initially pyrolyzed at 1000 °C, followed by hydrogen treatment at 600 °C to obtain C−1000 H_2_−600. The resultant C−1000 H_2_−600 possesses abundant active defect sites and oxygen functional groups, endowing it with high-rate capabilities (C−1000 H_2_−600 vs. commercial graphite: 223.98 vs. 198.5 mAh g^−1^ at 1 A g^−1^ with a capacity retention of about 72.79% vs. 58.05%, 196.97 vs. 109.1 mAh g^−1^ at 2 A g^−1^ for 64.01% vs. 31.91%), and a stable cycling life (205.5 mAh g^−1^ for 1000 cycles at 2 A g^−1^) for LIBs. This proves that as a simple moderator, hydrogen effectively tailors the microstructure and surface-active sites of carbon materials and transforms low-cost DQC into high-value advanced carbon anodes by a green and sustainable route to improve the lithium storage performance.

## 1. Introduction

The global energy crisis resulting from the unreasonable utilization of traditional fossil energy and severe environmental issues, such as the greenhouse effect, have triggered exploration of alternative sustainable, pollution-free energy sources and high-performance energy storage devices [1]. Rechargeable lithium-ion batteries (LIBs), representing one of the most important energy storage devices, have rapidly come to dominate the market in recent decades due to their high power and energy densities [2]. However, several factors have considerably influenced the widespread commercial application of LIBs, including expensive materials, poor high-rate performance, and complex synthetic conditions [1,3]. The current market price of graphite is exhibiting a gradual increasing trend. Commercial graphite anodes, as a crucial and mainstream material for LIBs, has a low theoretical capacity (372 mAh g^−1^) and poor rate performance for small interlayer spacing and less active sites, making them incapable of fully supporting the rapid diffusion of lithium-ion in LIBs [1,3]. Furthermore, graphite is traditionally synthesized by high pressure (graphitization temperature of 1200~1700 °C) [4] or an energy-intensive thermal process (Acheson process) at ~3300 K [5], which is a major factor influencing the high cost of graphite and its complex synthetic conditions. In addition to the cost, the low theoretical capacity and poor high-rate performance of graphite are considered major obstacles to the widespread adoption of electric vehicles (high-rate capability) [6,7]. Dry-quenched coke (DQC), an earth-abundant and low-cost byproduct of coal, plays an important role in the industry. However, DQC is currently only applied in low-added-value products. Hence, transforming DQC into high-value advanced carbon anodes by a simple green and sustainable route for LIBs to motivate the “trash to treasure” route may effectively alleviate the current issues associated with graphite. Recently, many coke-based carbon anode materials for LIBs, such as needle coke [8], pitch [9], and anthracite [10], have been successfully prepared from the low added-value by-products of coal, suggesting its potential applications in high-value advanced products. Although such high-value advanced products are prepared with simple and low-cost methods, they all still show poor high-rate performance in LIBs.

To improve the poor rate performance under fast-charging conditions, several approaches to modifying the graphite structure (increasing the interlayer distance, doping heteroatoms, and coating the surface) have been attempted to achieve highly active sites and larger interlayer spacing architecture with high-rate performance [11,12,13]. Tailoring defects is a very effective method to adjust the microstructure of electrode materials and regulate their energy storage performance [14]. Many researchers have demonstrated that extensive defects can significantly improve the electrochemical performance through theoretical and experimental methodologies [15]. One of the most effective strategies is to introduce heteroatoms into the carbon lattice framework, repeatedly demonstrating that they not only regulate the interlayer spacing to support fast-charging storage kinetics for alkali ions but produce rich active sites (vacancies, defects, and edge sites) for the adsorption of alkali ions to improve the capacity performance of alkali-ion batteries [16,17,18,19]. The introduction of heteroatoms and tailoring of defects can be realized simultaneously. Chen et al. prepared modified graphite by microwave irradiation of partially oxidized graphite, achieving an initial coulomb efficiency (ICE) of 40% and a reversible capacity of 370 mAh g^−1^ after 410 cycles, which is close to the theoretical capacity of 372 mAh g^−1^ [20]. Zou et al. designed an NPCS–1 sample by optimizing the intrinsic structure and surface functional groups, achieving excellent performance for lithium-ion capacitors with an appropriate N–to–O ratio [21]. Piedboeuf et al. proved that surface aldehyde (HC=O) groups can improve the Li^+^ ion storage capacity better than quinone (C=O) groups or hydroxyl (–OH) groups [22]. These studies proved that the type of oxygen functional group significantly affects the electrochemical performance because reversible redox-active sites rapidly absorb alkali ions to achieve high-rate performance [23]. DQC material contains a non-negligible amount of oxygen functional groups depending on precursors and synthesis procedures. Therefore, it is necessary to adjust the types of oxygen functional groups to improve electrochemical performance, especially the high-rate performance, owing to the poor rate performance under fast-charging conditions for graphite. Graphite consists of π-π stacking of layered hexagonal carbon via a weak van der Waals interaction, in which carbon atoms bond via sp^2^ (trigonal) hybridization to form a hexagonal pattern, and the hydrogen atom uses its one electron to form a C–H bond [24,25,26]. The hydrogen atom is the simplest free radical in carbon material, but as a moderator, it can drastically change the electronic state of the materials and simultaneously plays an important role in tailoring the type and content of non-negligible oxygen functional groups of carbon materials. Terakura’s and colleagues demonstrated the variation in edge terminations required for changes in chemical bonding and localized edge states, depending on the ratio of monohydrogen (–CH) to dihydrogen (–CH_2_) terminations [27]. Their forthcoming work further shows that oxygen atoms are mainly distributed in vacancy sites rather than the bulk region of graphene and that two carbon atoms next to the vacancy site are beneficial to form ether groups, along with –CH, –CH_2_, and –OH groups or –CH plus –OH groups for oxidized monovacancy under the hydrogen [28]. Therefore, hydrogen is a suitable tailoring moderator, and its influence on the physicochemical properties of DQC should be explored application in LIBs.

Herein, we used earth-abundant and low-cost DQC as a precursor to prepare low-crystalline carbon material using hydrogen as a simple green moderator for high-rate performance in LIBs. DQC was initially pyrolyzed at 1000 °C, followed by treatment with hydrogen as a simple green moderator at 600 °C to obtain the underdeveloped turbostratic graphical nanodomain (TGND) carbon material (C−1000 H_2_−600). The resultant C−1000 H_2_−600 is rich in defect sites, with numerous active oxygen functional groups. The C−1000 H_2_−600 anode displays a high-rate capability and a stable cycling life (205.5 mAh g^−1^ for 1000 cycles at 2 A g^−1^) in LIBs. This proves that the microstructure of carbon materials can be effectively tailored by hydrogen as a simple green moderator, and low-value DQC can be turned into high-value advanced carbon anodes to modify lithium storage performance.

## 2. Materials and Methods

### 2.1. Materials Preparation

Dry-quenched coke (DQC) powder raw material was obtained from Zhengzhou city, Henan province, China (purchased on www.taobao.com/, 15 October 2019). The DQC powders were ball-milled for 12 h at a speed of 400 rpm in a planetary ball mill and washed with 1 M HCl, 5% HF, and distilled water to remove undesired ions. The DQC powders were thoroughly dried at 110 °C for 24 h in a blast drying oven. Then, the DQC powders were initially carbonized at 1000 °C in a high-temperature tube furnace (named C−1000) for 2 h at a heating rate of 5 °C min^−1^ under an argon atmosphere (20 mL/min). After the initial carbonization, the collected materials were further calcined at 600 °C in an argon atmosphere (named C−1000 Ar−600, as a control sample) and at 600 °C in a mixing atmosphere with 10 wt.% hydrogen and 90% argon (named C−1000 H_2_−600). A detailed schematic diagram of the preparation process for the three samples is shown in Figure S1, and the three sample names and their parameters are shown in Table 1. Finally, the above materials were stored in dry glass vessels for later electrode preparation and related characterization tests.

### 2.2. Material Characterization

Powder X-ray diffraction (XRD, D/max-2500 pc, Rigaku, Tokyo, Japan) at 40 kV with Cu Kα radiation (*λ* = 1.5406 Å), field emission scanning electron microscopy (FESEM, JEOL JSM-7600F, Tokyo, Japan), high-resolution transmission electron microscopy (HRTEM, JEOL JEM-2100F, Tokyo, Japan) with energy-dispersive X-ray spectroscopy (EDS), Raman spectroscopy (Horiba LabRAM HR Evolution, laser beam 532 nm), and N_2_ adsorption/desorption isotherms by Brunauer–Emmett–Teller (BET) method (Micrometrics ASAP 2050, OR, USA) at 77 K were used to analyze the morphologies and characteristic information of the abovementioned materials. The element chemical states were traced via Fourier transform infrared spectroscopy (FTIR, Nicolet iS10, Thermo Fisher Scientific, Waltham, MA, USA), X-ray photoelectron spectroscopy (XPS, Thermo Scientific ESCALAB 250Xi, Waltham, MA, USA), Ar^+^ ion sputtering, and electron paramagnetic resonance (EPR) spectroscopy (BrukerBioSpin, a Bruker super-high Q resonator ER4122SHQE, Rheinstetten, Germany). Thermogravimetric analysis (TGA) (NETZSCH STA449F3) was carried out under an N_2_ atmosphere.

### 2.3. Electrochemical Testing

Working electrodes were prepared by uniformly casting a mixture slurry of 80 wt.% C−1000 H_2_−600 (C−1000, C−1000 Ar−600), 10 wt.% Ketjen black, and 10 wt.% polyvinylidene fluoride in *N*-methyl-2-pyrrolidinone on copper and dried at 110 °C for at least 12 h in a vacuum oven. Three electrodes were prepared with mass loadings of approximately 1.0–1.3 mg cm^−2^. 2032-type coin cells were assembled with the working electrodes, with pure Li foil as the counter electrode, single-layer polypropylene filters (Celgard 2320) as the separator, and the electrolyte (1 M LiPF_6_ in diethyl carbonate, ethylene carbonate, and dimethyl carbonate with a volume ratio = 1:1:1) in an argon-filled glove box (MIKROUNA). LAND-CT2001A multichannel battery testing systems (LAND Electronic Co., Wuhan, China) were used to record galvanostatic discharge/charge curves and galvanostatic intermittent titration technique (GITT) curves. Cyclic voltammetry (CV) curves were obtained at varying sweeping rates on a CHI 750A workstation (Chenhua, Shanghai).

## 3. Results and Discussion

**Figure 1:**
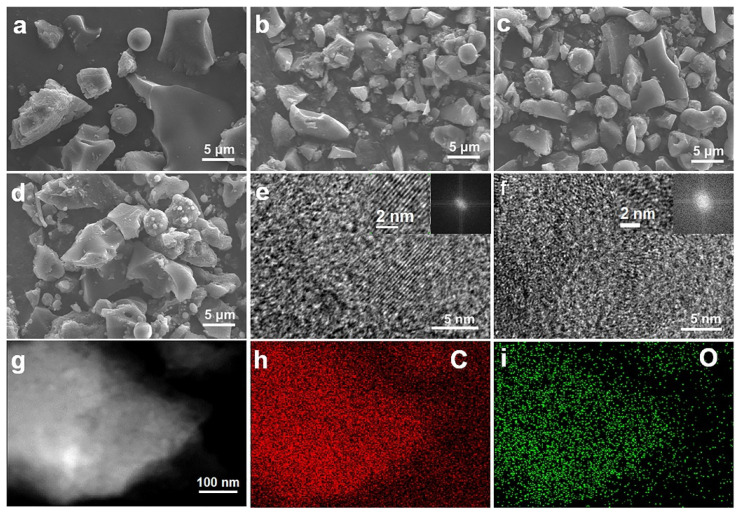
SEM images of (**a**) DQC, (**b**) C−1000, (**c**) C−1000 Ar−600, and (**d**) C−1000 H_2_−600. HRTEM images of (**e**) C−1000 Ar−600, and (**f**) C−1000 H_2_−600; inset: partial magnified images and FFT images. (**g**) TEM image of C−1000 H_2_−600. EDS mapping of C−1000 H_2_−600 with (**h**) C element and (**i**) O element.

DQC has the lowest price and highest carbon yield at 1000 °C compared with other common carbonaceous precursors (Table S1). The carbon yield curve of DQC obtained by thermogravimetric analysis (TGA) is displayed in Figure S2. The morphologies of DQC were characterized before and after carbonization by scanning electron microscope (SEM) (Figure 1a–d) (partially magnified SEM images are shown in Figure S3). The SEM images show mostly irregular particles and few microspheres about 5~10 μm in size without significant differences. As shown in Figure S3a–d, the surface of the materials present with an increasingly smooth with surface, with few tiny particles attached. The microstructures of the carbon anode materials determine the electrochemical storage performance in LIBs. Therefore, we used transmission electron microscopy (TEM) to further explore the microstructure of the tailored carbon materials, which showed laminar sheet-like morphologies (C−1000 Ar−600 in Figure S4a,c, C−1000 H_2_−600 in Figure S4b,d). C−1000 Ar−600 displayed a significant lattice fringe with locally developed long-range ordered turbostratic graphical nanodomains (TGNDs), indicating its superior graphitic structure (Figure 1e), whereas C−1000 H_2_−600 displayed underdeveloped TGNDs in high-resolution TEM (HRTEM) images (Figure 1f). C−1000 H_2_−600 showed more defect edge sites and voids (Figure 1f) compared to C−1000 Ar−600 (Figure 1e) because hydrogen tailors the π-π stacking of a layered hexagonal pattern of graphite [29,30,31]. The rich defect edge sites and voids of C−1000 H_2_−600 can support faster charge storage kinetics of lithium-ion due to the good electrode/electrolyte wettability, which is beneficial to improve the high-rate performance [32,33]. Energy-dispersive X-ray spectroscopy (EDS) mapping images demonstrate that the O atom is uniformly distributed in the carbon crystal lattice of C−1000 H_2_−600 (Figure 1g–i), and FTIR spectra further show that C−1000 H_2_−600 has more C=O/COO/C–O groups and C-H group than C−1000 Ar−600 and C−1000 (Figure S5).

**Figure 2:**
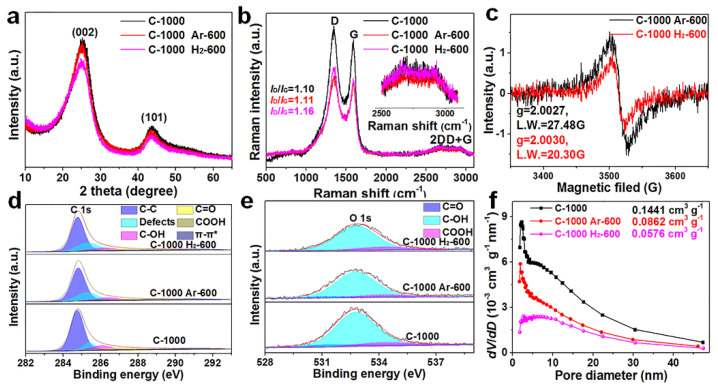
(**a**) XRD patterns of the three samples. (**b**) Raman spectra of the three samples. (**c**) EPR spectra of the three samples. XPS spectra of (**d**) C 1s and (**e**) O 1s of the three samples. (**f**) Pore size distribution of the three samples.

To further study the microstructure and physicochemical properties of the three materials, XRD, Raman, EPR, XPS, and BET characterization tests were performed. The three materials all showed two broad diffraction peaks: (002) at about 25.0° and (101) at about 43.5°. The average interlayer spacing of 0.357 nm for C−1000 H_2_−600 (the (002) peak is 25.00°) is larger than that of commercial graphite (0.335 nm) as calculated by the Bragg equation, which is slightly larger than that of the other two materials (C−1000 Ar−600 (the (002) peak is 25.14°): 0.354 nm; C−1000 (the (002) peak is 25.28°): 0.352 nm) (Figure 2a). The (002) peak intensity and the peak width at half height of C−1000 Ar−600 and C−1000 Ar−600 are almost the same and are higher and narrower than that of C−1000 H_2_−600, implying fewer parallel laminated graphite layers in C−1000 H_2_−600 (Figure 1f). The weaker (101) peak intensity shows a decreased degree of graphitization due to the tailored π-π stacking of the layered hexagonal pattern of graphite in C−1000 H_2_−600 [29,30,31]. The degree of graphitization for amorphous carbon can be evaluated by the empirical *R*-factor value [34]. The higher *R*-factor value of C−1000 Ar−600 (4.12) (C−1000, 4.20) indicates a higher degree of graphitization compared to C−1000 H_2_−600 (3.39), which is in agreement with the HRTEM result. The Raman spectra of the three materials all display two broad peaks, namely D bands (about 1350 cm^−1^) and G bands (about 1590 cm^−1^), as shown in Figure 2b. The *I*_D_/*I*_G_ ratio of 1.16 for C−1000 H_2_−600 is larger than that of C−1000 Ar−600 (1.11) and C−1000 (1.10), implying numerous defect sites in C−1000 H_2_−600 [35]. Moreover, the intense second index characteristic peaks of 2D and D+G bands also indicate more defect sites in the three materials [35]. EPR spectra were utilized to explore the delocalized unpaired electrons shown in Figure 2c. The higher *g* value of 2.0030 from the Lorentzian EPR line for C−1000 H_2_−600 relative to 2.0027 for C−1000 Ar−600 shows a strong hydrogen-doping effect. The lower linewidth (LW) of 20.30 G for C−1000 H_2_−600 relative to 27.48 G for C−1000 Ar−600 indicates more localized unpaired electrons resulting from hydrogen doping and underdeveloped TGNDs. According to the EPR spectra, the carbon matrix of C−1000 H_2_−600 is divided into small TGNDs with rich defect sites [36], and the rich delocalized unpaired electrons result in faster charge storage kinetics for lithium-ion to achieve high-rate performance in LIBs. Based on the localized electrons and defect site situations, we further explored the content, species, and binding states of oxygen functional groups for the three materials based on the XPS spectra shown in Figure 2d,e. The deconvoluted C 1s spectra (Figure 2d) of the three samples are divided into six subpeaks, namely a C−C peak at 284.5 eV, a defect peak (sp^3^ hybridized carbon peak) at 285.4 eV, a C−OH peak at 286.3 eV, a C=O peak at 287.8 eV, a COOH peak at 288.9 eV, and a π-π* transition peak at 290–294 eV [37,38,39,40]. Similarly, the deconvoluted O 1s spectra (Figure 2e) of the three samples are divided into three subpeaks, namely a C=O peak at 531.3 eV, a C−OH peak at 532.8 eV, and a COOH peak at 534.2 eV. C−1000 H_2_−600 has a lower oxygen content than C−1000 Ar−600 and C−1000 (Table S2), implying a strong hydrogen-tailoring effect and partial removal of oxygen. The higher (defect peak, C−OH, COOH) and lower (C=O, π-π*) contents in C−1000 H_2_−600 relative to those C−1000 Ar−600 and C−1000, respectively (Tables S3 and S4), are ascribed to a strong hydrogen-tailoring effect; these results are consistent with the EPR spectra. The C−OH content in C−1000 Ar−600 is slightly lower than that of C−1000, as the epoxy group was thermally removed at annealing temperatures above 260 °C [41]. Many researchers have proven that the contributions to surface hydrophilicity of oxygen functional groups occur in the following order: COOH > C−OH > C=O [42,43,44]. Therefore, C−1000 H_2_−600 can produce rich reversible redox-active sites and wettability between the electrode/electrolyte to improve the rate performance [45]. Finally, we explored the porosity and special surface areas of the three materials contributed by the N_2_ adsorption/desorption isotherms. C−1000 H_2_−600 has the smallest special surface area of 28.09 m^2^ g^−1^ and the largest average pore size of 8.20 nm compared with the other two materials (detailed information in Table S5) due to hydrogen tailoring of the edge of the hole, also leading to the collapse of the carbon structure and disorder of the carbon layer. All three materials display typical type-IV isotherms (Figure S6) and a pore size distribution ranging from 1.7 to 50 nm, with both micropores and mesopores (Figure 2f). The smallest special surface area of C−1000 H_2_−600 can mitigate parasitic reactions to form a thin solid electrolyte interphase (SEI) film to improve the reversible long cycle stability, and the larger average pore size can support faster lithium-ion diffusion kinetics to achieve high-rate performance in LIBs [45,46,47,48]. Therefore, the hydrogen tailoring in C−1000 H_2_−600 can produce rich COOH and OH groups and increase numerous active defect sites to improve the high-rate performance [45,46,47].

**Figure 3:**
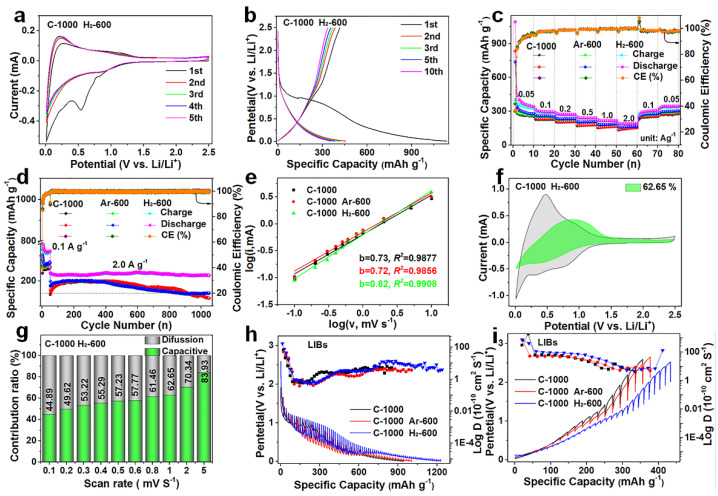
Electrochemical performance characterization of the three electrode materials in LIBs. (**a**) CV curves of C−1000 H_2_−600 at 0.2 mV s^−1^ under the potential range of 0.01−2.5 V. (**b**) GCD curves of the three electrode materials at 0.1 A g^−1^ under the potential range of 0.01−2.5 V. (**c**) Cycling performance of the three electrode materials at varying densities. (**d**) Cycle life for 1000 cycles of the three electrode materials at 2 A g^−1^. (**e**) Plots between log(*v*) and log(*i*) for the *b* value at various scan rates of the three electrode materials. (**f**) Capacitive contribution of C−1000 H_2_−600 (green) at 1 mV s^−1^. (**g**) Capacitive contribution of C−1000 H_2_−600 (green) at various scan rates. (**h**,**i**) GITT curves and Li^+^ diffusion coefficient of the three electrode materials.

According to the structural characteristics described above, C−1000 H_2_−600, C−1000 Ar−600, and C−1000 are endowed with excellent electrochemical properties; therefore, their lithium storage performances as anodes were studied through a series of electrochemical tests. The first cyclic voltammetry (CV) curves of C−1000 H_2_−600 (Figure 3a), C−1000 (Figure S7a), and C−1000 Ar−600 (Figure S7b) all show irreversible narrow current peaks at about 0.5 V at a scanning rate of 0.2 mV s^−1^, which is indicative of SEI film formation for the irreversible entrapment of lithium ions on the surfaces of the three electrode materials [16,49,50]. The subsequent CV curves of the three electrode materials progressively overlap, indicating a decrease in their capacities during the several initial cycles and becoming increasingly electrochemical stable. The lithium-ion storage capabilities of C−1000 H_2_−600 (Figure 3b), C−1000 (Figure S7c), and C−1000 Ar−600 (Figure S7d) were evaluated using galvanostatic charge/discharge (GCD) analysis; their initial discharge/charge capacities and ICEs are displayed in Table S6. C−1000 H_2_−600 shows higher initial discharge/charge capacities than C−1000 Ar−600 and C−1000 in LIBs (Table S6). The typical sloping GCD curves of C−1000 H_2_−600 show the lowest potentials during the charging process and the highest potentials during the discharge process at the same capacities (Figure S8), which are contributed to the microstructural active defect sites and the rich active functional groups to liberate capacity contributions [51,52]. Figure 3c shows the rate performances of the three electrode materials at various current densities from 0.05 to 2 A g^−1^; the discharge special capacities of C−1000 H_2_−600 in LIBs (383.52, 307.70, 282.48, 248.32, 223.98, and 196.97 mAh g^−1^ from 0.05 to 2 A g^−1^) are significantly higher than those of C−1000 Ar−600 and C−1000 at same current density (detailed information in Table S7**)**. When cycled at 0.05 A g^−1^, their capacities all almost recover to the initial level, indicating their sufficient rate capability. It is obvious that the discharge capacities of C−1000 H_2_−600 at the high current densities of 1 and 2 A g^−1^ are significantly higher than those of commercial graphite in LIBs, demonstrating the excellent high-rate performance of C−1000 H_2_−600 (C−1000 H_2_−600 vs. commercial graphite: 223.98 vs. 198.5 mAh g^−1^ at 1 A g^−1^ with a capacity retention of about 72.79% vs. 58.05%, 196.97 vs. 109.1 mAh g^−1^ at 2 A g^−1^ for 64.01% vs. 31.91%) (Table S7 and Figure S9). The high-rate performances at 1 and 2 A g^−1^ are almost the highest among the recently reported literature on coal-based carbon anodes (Table S8) [9,53,54,55,56,57,58,59]. The corresponding GCD curves of the rate performances for the three electrode materials are shown in Figure S10a–c, respectively. The cycling performances of the three electrode materials were tested at 0.1 A for 50 cycles and 1000 cycles at 2 A g^−1^ (Figure 3d). After 50 cycles, C−1000 H_2_−600 delivers a reversible capacity of 296.15 mAh g^−1^, which is higher than that of 273.43 mAh g^−1^ for C−1000 Ar−600 and 257.26 mAh g^−1^ for C−1000. In the subsequent 1000 cycles, C−1000 H_2_−600 still delivers a stable reversible capacity of 205.53 mAh g^−1^, with a high-capacity retention rate of 87.71% (234.33 mAh g^−1^ in the 51st cycle at 2 A g^−1^), corresponding to a 0.0288% capacity fading per cycle in LIBs (Figure 3d), which is than that of C−1000 Ar−600 (151.91 mAh g^−1^, 76.24%, 0.047%) and C−1000(131.37 mAh g^−1^, 79.95%, 0.033%). The GCD curves of the cycling performances for the three electrode materials are shown in Figure S10d–f, respectively. The excellent high-rate performance and the capacity retention rate of C−1000 H_2_−600 in LIBs are contributed by its underdeveloped TGNDs, larger average pore size, microstructural active defect sites, and rich active functional groups to effectively support faster lithium-ion diffusion kinetics [45,46,47,48,60].

To further explore the fast lithium-ion diffusion kinetics, the relationship between the peak current (*i*) and scan rate (*v*) is studied and described as Equation (1) [61,62].

(1) $$i=av^{b}\;and\;\log\left(i\right)=b\times \log\left(v\right)+\log\left(a\right)$$

The process is controlled by an absolute capacitance process when the *b* value equals 1.0, whereas a *b* value of 0.5 indicated the electrochemical response by the diffusion-controlled process [63,64]. The lithium-ion storage kinetics behaviors of the three electrode materials were calculated at varying CV scan rates from 0.1 to 5.0 mV s^−1^ in LIBs (Figure S11). C−1000 H_2_−600 shows a higher *b* value of 0.82 for anodic peaks than that of 0.72 for C−1000 Ar−600 and 0.73 for C−1000 in LIBs (Figure 3e). The capacitance contributions were further quantified according to Equation (2) based on Dunn’s method [65,66].

(2) $$i\left(V\right)=k_{1}v+k_{2}v^{1/2}$$

The capacitance contribution rates of C−1000 H_2_−600, C−1000, and C−1000 Ar−600 can reach 62.65% (Figure 3f), 61.60% (Figure S12a), and 57.72% (Figure S12b), respectively, at a scan rate of 1.0 mV s^−1^. The detailed calculated results of C−1000 H_2_−600 at different CV scans rates are shown in Figure 3g; the capacitance contribution rates gradually increase from 44.89 to 83.93% within scan rate range of 0.1 to 5.0 mV s^−1^. The same trends are shown in C−1000 (43.80 to 82.82%, Figure S12c) and C−1000 Ar−600 (42.23 to 80.75%, Figure S12d). Higher *b* values and higher capacitive contributions of C−1000 H_2_−600 are mainly attributed to its underdeveloped TGNDs, large average pore size, microstructural active defect sites, and rich active functional groups to effectively support faster lithium-ion diffusion kinetics [45,46,47,48,60]. Furthermore, the lithium-ion reaction kinetics of the three electrode materials during the discharge/charge process were explored in depth by GITT [67]. As shown in Figure 3h,i, C−1000 H_2_−600 shows relatively higher the diffusion coefficient (*D_Li+_*) values to effectively support faster lithium-ion diffusion kinetics in LIBs compared to C−1000 Ar−600 and C−1000 (SEM images of the electrode thickness (*L*) for the three electrode materials are shown in Figure S13). Here, *D_Li+_* was calculated according to the following formula.

(3) $$D_{Li^{+}}=\frac{4L^{2}}{\pi \tau }{\left(\frac{\Delta E_{s}}{\Delta E_{t}}\right)}^{2}$$

where *τ* (s) is the relaxation time, *L* (cm) is the length of Lithium-ion diffusion routes, ∆*E_s_* is the variation of potential (*V*) arising from the current pulse, and ∆*E_t_* is the variation of potential (V) in the process of the galvanostatic charging/discharging. The higher *D_Li+_* values of C−1000 H_2_−600 are mainly the result of its large average pore size and its numerous active defect sites and functional groups [46,47,48]. The lithium-ion diffusion kinetics of the three electrode materials before cycling and after cycling can be characterized by the electrochemical impedance spectra (EIS), explaining the excellent electrochemical performance. According to analysis of the Nyquist plots, C−1000 H_2_−600 lowest the smallest charge-transfer resistance (*R*_ct_) and ohmic contact resistance (*R*_s_) and the highest sloping line compared to C−1000 Ar−600 and C−1000 before cycling and after cycling in LIBs (Figure S14; detailed information in Table S9), which is consistent with the *D_Li+_* value. This proves that C−1000 H_2_−600 has the lowest ion diffusion impedance, providing benign ion diffusion and reaction kinetics to achieve high-rate performance. The *R*_ct_ values of the three materials are all higher after cycling than that before cycling in terms of SEI film formation [68,69]. The above results demonstrate that C−1000 H_2_−600 has fast lithium-ion diffusion kinetics to support high-rate performance in LIBs.

**Figure 4:**
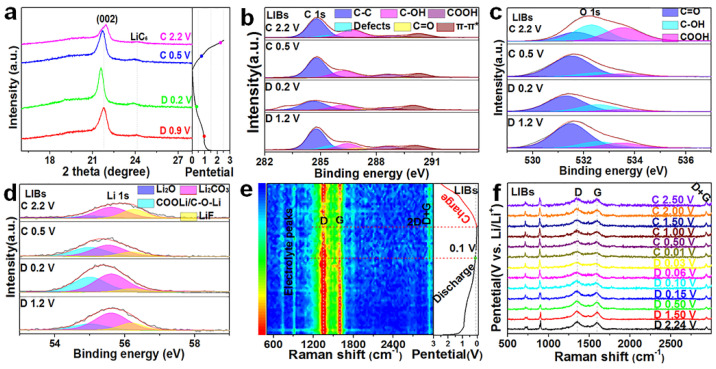
Electrochemical performance characterization of the three electrode materials in LIBs. (**a**) Ex situ XRD of C−1000 H_2_−600 at varying discharge/charge voltages. Ex situ XPS spectra of (**b**) C 1s, (**c**) O 1s, and (**d**) Li 1s in LIBs for C−1000 H_2_−600 at varying discharge/charge voltages. (**e**) In situ Raman mapping and (**f**) the corresponding spectra of C−1000 H_2_−600 during the first discharge/charge process.

We first studied the microstructural stability of C−1000 H_2_−600 according to the variation tendency of the (002) peak by ex situ XRD during the discharging/charging process in LIBs (Figure 4a). The ex situ XRD patterns show an obvious left-shifting trend of the (002) peak and the generation of LiC_6_ during the discharging process, as well as a tendency to return to the initial position and weakening of LiC_6_ after charging, suggesting good structural reversibility in the TGNDs for lithium-ion batteries [51]. Then, we analyzed the changes in the composition of surface functional groups of C−1000 H_2_−600 during the discharging/charging processes and explored the possible reaction mechanism. C−1000 H_2_−600 has 4.05% oxygen functional groups according to the XPS spectrum. Enhanced capacitance depends on numerous HC=O and COOH groups for more negative electrons to provide more orbital distributions and C=O and –OH groups to improve the capacity and high-rate performance [22,70]. The possible faradaic redox reactions are described as follows:

HC=O + Li^+^ + e^−^ ↔ HC−O−Li (4)

COOH + Li^+^ + e^−^ ↔ HO−CO−Li (5)

To fully understand the roles of oxygen functional groups of C−1000 H_2_−600, ex situ XPS and in situ Raman measurements were performed to reveal the underlying mechanisms. Table S10 displays the element percentages of C−1000 H_2_−600 based on total XPS spectra at varying discharge/charge voltages; the percentage of Li 1s increases and decreases, representing lithium-ion storage processes during the discharging/charging process, respectively. Although the deconvoluted C 1s and O 1s spectra are all divided into subpeaks of C=O, C−OH, and COOH, electrolytes containing large numbers of oxygen functional groups compared to relatively lower oxygen functional groups (<5 at %) have a larger deviation due to the complex decomposition products from the electrolyte; therefore, records were made without in-depth analysis (Figure 4b and Table S11 for the deconvoluted C 1s; Figure 4c and Table S12 for the deconvoluted O 1s; D, discharge; C, charge). The decreasing percentage of defects from the deconvoluted C 1s indicates the adsorption of lithium ions during the discharging process, and the increasing percentage of defects indicates the desorption process of lithium ions during the charging process. In the Li 1s spectrum of C−1000 H_2_−600 was divided into four deconvoluted peaks, namely Li_2_O, COOLi/COLi, Li_2_CO_3_, and LiF, were divided; an increasing percentage of COOLi/COLi implies the forward reaction processes represented by Equations (3) and (4) during the discharging process, whereas the decreasing percentage of COOLi/COLi is a reverse reaction processes during the charging process (Figure 4d for deconvoluted Li 1s; detailed proportions of groups are presented in Table S13) [71]. In situ Raman mapping (Figure 4e) and Raman spectra (Figure 4f) of C−1000 H_2_−600 were used to reveal the potential mechanism of lithium-ion adsorption on the microstructural active defect sites and the rich active function groups, as well as that of de-/intercalation into the TGND layers during the discharge/charge process in LIBs [72]. The position and intensity of the D band (near 1350 cm^−1^) and G band (near 1590 cm^−1^) are affected by the adsorption/desorption and de-/intercalation of lithium ions into the carbon framework. The decreasing intensity of the D band from the open-circuit voltage (OCV) to 0.01 V is caused by the confinement of the breathing motion of sp^2^ atoms in the rings at the edge planes, and the intensity of the G band is not only gradually weakened from OCV to 0.1 V, but the peaks are also slightly red-shifted (Figure 4e,f), preventing the occupation of the active defect sites and functional groups of C−1000 H_2_−600 by lithium ions [71]. The decreasing intensity of the G band from 0.1 to 0.01 V in LIBs is caused by the weakening of resonance for the intercalation of lithium ions into the underdeveloped TGNDs to achieve the lithiation process [71,72]. Finally, the recovery of the original D and G bands for C−1000 H_2_−600 after the charging process demonstrates its reversibility.

## 4. Conclusions

In this work, we used earth-abundant and low-cost DQC as the precursor to prepare low-crystalline carbon material with hydrogen as a simple green moderator for high-rate performance in LIBs. DQC was initially pyrolyzed at 1000 °C, followed by treatment with hydrogen as a simple green moderator at 600 °C to obtain the crystalline carbon material (C−1000 H_2_−600). C−1000 H_2_−600 showed mostly irregular particles and few microspheres about 5~10 μm in size with underdeveloped TGNDs. As a result, the prepared C−1000 H_2_−600 is rich in defect sites, with numerous active oxygen functional groups, as demonstrated by XRD, Raman, and XPS images. The C−1000 H_2_−600 anode displays a high rate capability and a stable cycling life (205.5 mAh g^−1^ for 1000 cycles at 2 A g^−^^1^) in LIBs. This proves that the microstructure of carbon materials can be effectively tailored by hydrogen as a simple green moderator and that low-value DQC can be turned into high-value advanced carbon anodes to achieve satisfactory lithium storage performance.

## Figures and Tables

**Table 1:** Sample names and their parameters.

Material	First Calcination Temperature (°C)	First Calcination Atmosphere	Second Calcination Temperature (°C)	Second Calcination Atmosphere
C−1000	1000	100% argon	----	----
C−1000 Ar−600	1000	100% argon	600	100% argon
C−1000 H_2_−600	1000	100% argon	600	10 wt.% hydrogen and 90% argon

## Data Availability

The data presented in this study are available upon request from the corresponding author.

## Supplementary Materials

The following supporting information can be downloaded at: https://www.mdpi.com/article/10.3390/nano12193530/s1, Table S1: Comparison of price and carbon yield at 1000 °C between DQC and common carbonaceous precursors; Figure S1: Schematic illustration of the preparation of C−1000, C−1000 Ar−600, and C−1000 H_2_−600; Figure S2: TGA profile of DQC under a nitrogen atmosphere; Figure S3: SEM images of (a) DQC, (b) C−1000, (c) C−1000 Ar−600, and (d) C−1000 H_2_−600; Figure S4: TEM images of (a, c) C−1000 Ar−600 and (b, d) C−1000 H_2_−600; Figure S5: FTIR spectra of C−1000, C−1000 Ar−600, and C−1000 H_2_−600; Table S2: XPS element contents of C−1000, C−1000 Ar−600, and C−1000 H_2_−600 samples; Table S3: XPS carbon bonding analysis of the three samples; Table S4: XPS oxygen bonding analysis of the three samples; Figure S6: N_2_ adsorption/desorption isotherms of C−1000, C−1000 Ar−600, and C−1000 H_2_−600 samples; Figure S7: CV curves of (a) C−1000 and (b) C−1000 Ar−600 at 0.2 mV s^−1^ under the potential range of 0.01~2.5 V; galvanostatic charge–discharge of (c) C−1000 and (d) C−1000 Ar−600 at 0.05 A g^–1^ in LIBs; Table S6: Initial discharge/charge capacities and initial coulombic efficiency of the three materials at 0.05 A g^−1^; Figure S8: Galvanostatic charge–discharge of C−1000, C−1000 Ar−600, and C−1000 H_2_−600 at (a) 0.1 A g^–1^ and (b) 0.2 A g^–1^ in LIBs; Table S7: Discharge capacities (from the fifth cycle at the corresponding current density) of the three materials at varying current densities in LIBs (results of the commercial graphite were obtained by our group); Figure S9. Rate performance of graphite; Table S8: Comparison of C−1000 H_2_−600 versus recently reported coal-based carbonaceous anodes for LIBs; Figure S10: Galvanostatic charge–discharge of (a, d) C−1000, (b, e) C−1000 Ar−600, and (c, f) C−1000 H_2_−600 at varying current densities in LIBs; Figure S11: CV curves of (a) C−1000, (b) C−1000 Ar−600, and (c) C−1000 H_2_−600 in LIBs at various scan rates under the potential range of 0.01~2.5 V; Figure S12: Capacitive contribution ratios (green) of the capacitive process for (a) C−1000 and (b) C−1000 Ar−600 at a scan rate of 1 mV s^−1^; Capacitive charge-storage contribution of (c) C−1000 and (d) C−1000 Ar−600 at various scan rates from 0.1 to 5 mV s^−1^ in LIBs; Figure S13: SEM images of the electrode thickness of (a) C−1000, (b) C−1000 Ar−600, and (c) C−1000 H_2_−600 on Cu foil; Figure S14: Electrochemical impedance spectra of C−1000, C−1000 Ar−600, and C−1000 H_2_−600 (A) before cycling and (B) after 1050 cycles; Table S9: Electrochemical impedance spectra of C−1000, C−1000 Ar−600, and C−1000 H_2_−600 before cycling and after cycling (1050 cycles); Table S10: XPS element contents of C−1000 H_2_−600 samples at different stages of discharge to 1.20 V and 0.20 V, charge to 0.50 V and 2.2 V states in LIBs; Table S11: Carbon bonding analysis of the C−1000 H_2_−600 samples at different stages of discharge to 1.20 V and 0.20 V, charge to 0.50 V and 2.2 V states in LIBs; Table S11: Carbon bonding analysis of the C−1000 H_2_−600 samples at different stages of discharge to 1.20 V and 0.20 V, charge to 0.50 V and 2.2 V states in LIBs; Table S12: Oxygen bonding analysis of the C−1000 H_2_−600 samples at different stages of discharge to 1.20 V and 0.20 V, charge to 0.50 V and 2.2 V states in LIBs; Table S13: Lithium bonding analysis of the C−1000 H_2_−600 samples at different stages of discharge to 1.20 V and 0.20 V, charge to 0.50 V and 2.2 V in LIBs. References [9,53,54,55,56,57,58,59] are cited in the Supplementary Materials.
