# Supplementary material for: Hydrogen Stabilization and Activation of Dry-Quenched Coke for High-Rate-Performance Lithium-Ion Batteries
Source: Nanomaterials (Basel). 2022 Oct 9;12(19):3530. doi: 10.3390/nano12193530 (PMC9565598; doi:10.3390/nano12193530)
Supplement: Supplementary file 1 [file nanomaterials-12-03530-s001.zip › nanomaterials-1960898-supplementary.pdf]

nanomaterials

MDPI

Supporting Information

Hydrogen Stabilization and Activation of Dry Quenching Coke for High Rate Performance Lithium-ion Batteries

Decai Qin [a], Fei Huang [a], Guoyin Zhu [b,]* and Lei Wang [a,]*

[a]School of Chemistry and Materials Science, Nanjing Normal University, Nanjing 210023, China
* Correspondence: lei_wang@njnu.edu.cn (L. Wang)

[b]Institute of Advanced Materials and Flexible Electronics (IAMFE), School of Chemistry and Materials Science, Nanjing University of Information Science & Technology, Nanjing 210044, China
* Correspondence: gyzhu@nuist.edu.cn (G. Zhu)

Table S1. Comparison of price and carbon yield at 1000 °C between CDQ and some common carbonaceous precursors.[a]

Carbon precursors	DQC	Pitch	Sucrose	Lignin	Starch	Cellulose	Phenolic Resin	Natural graphite	Artificial graphite
Price ($/ton)	130	300	400	450	500	1000	2000	3000~8000	5000~8000
Carbon production rate(%)	>85	~56	<10	~43	<10	<10	~47	---	---

[a] http://alibaba.com/.15th October, 2019

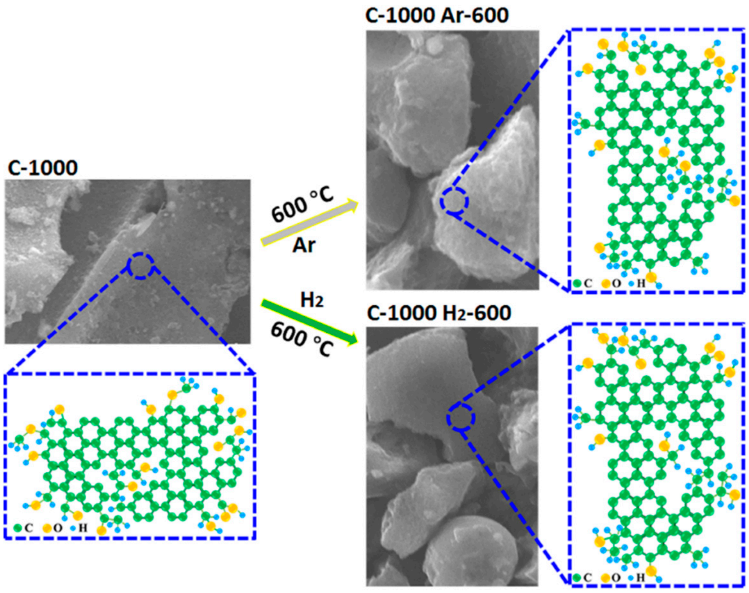

Figure S1. Schematic illustration of the preparation of C-1000, C-1000 Ar-600 and C-1000 H2-600.

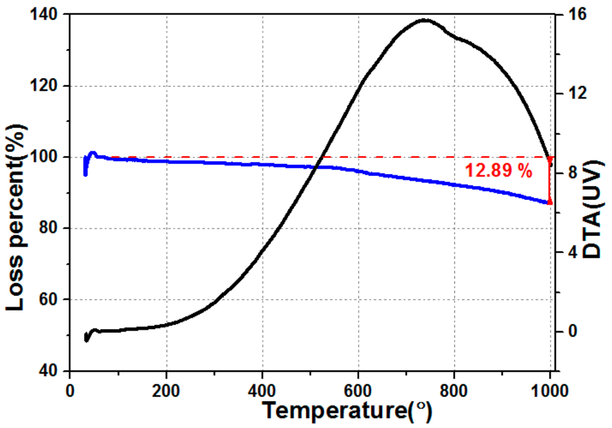

Figure S2. TGA profile of the DQC under a nitrogen atmosphere.

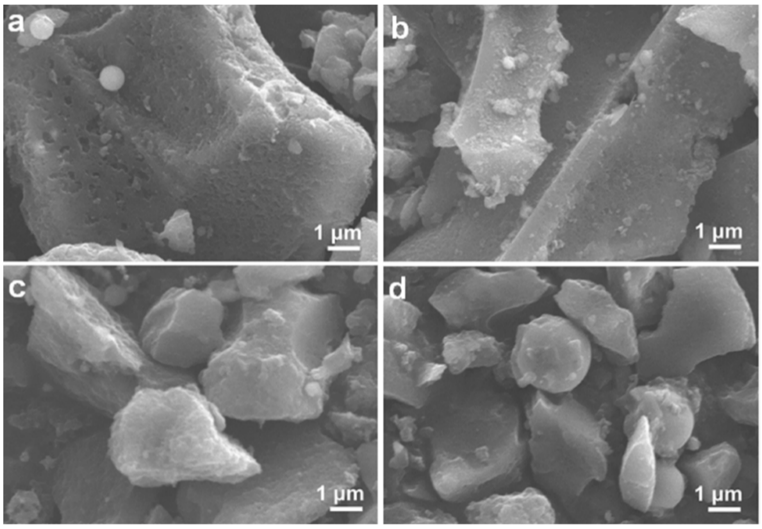

Figure S3. SEM images of (**a**) DQC, (**b**) C-1000, (**c**) C-1000 Ar-600, (**d**) C-1000 H_2-600.

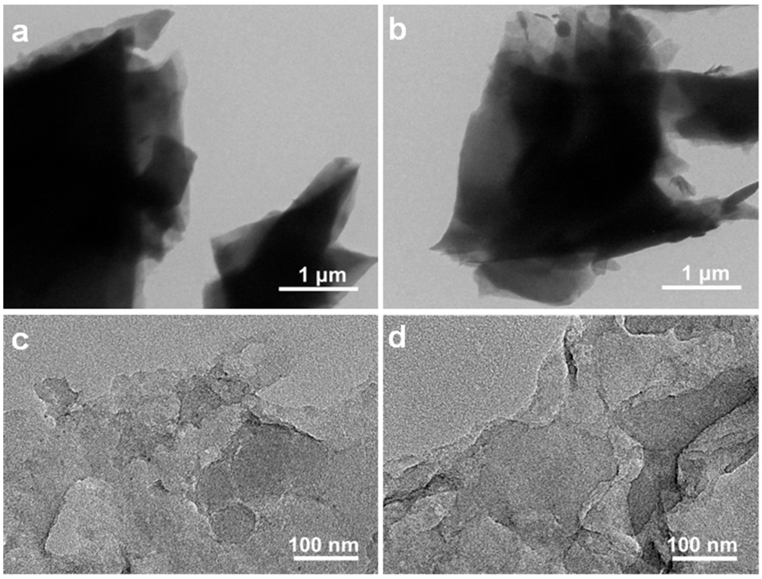

Figure S4. TEM images of (**a**,**c**) C-1000 Ar-600, (**b**,**d**) C-1000 H_2-600.

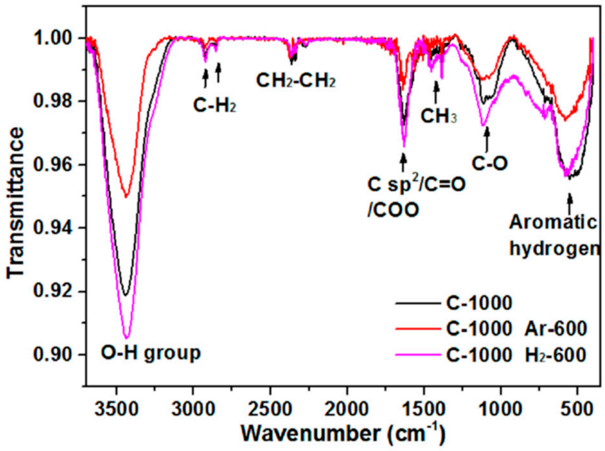

Figure S5. FTIR spectra of C-1000, C-1000 Ar-600 and C-1000 H_2-600

Table S2. The element contents from XPS of the C-1000, C-1000 Ar-600, C-1000 H_2-600 samples.

Elements	Percentage (at %)		
	**C-1000**	**C-1000 Ar-600**	**C-1000 H_2-600**
C	95.26	95.29	95.95
O	4.74	4.71	4.05

Table S3. The carbon bonding analysis of XPS of the three samples.

Carbon Bonding	Percentage (at %)		
	**C-1000**	**C-1000 Ar-600**	**C-1000 H_2-600**
C-C	54.82(57.55%)	55.5(58.24%)	55.36(57.70%)
Defects	18.57(19.49%)	18.89(19.83%)	21.42(22.33%)
C-OH	6.50(6.81%)	6.12(6.42%)	7.44(7.75%)
C=O	4.49(4.72%)	4.67(4.90%)	3.45(3.60%)
COOH	3.45(3.62%)	3.53(3.71%)	3.95(4.11%)
π-π*	7.43(7.80%)	6.58(6.91%)	4.33(4.51%)

Table S4. The oxygen bonding analysis of XPS of the three samples.

Oxygen Bonding	Percentage (at%)		
	**C-1000 (4.74)**	**C-1000 Ar-600 (4.71)**	**C-1000 H_2-600 (4.05)**
C=O	0.41(8.67%)	0.42(8.93%)	0.14(3.47%)
C-OH	3.93(82.79%)	3.82(81.01%)	3.37(82.93%)
COOH	0.40(8.54%)	0.47(10.06%)	0.54(13.30%)

Table S5. Structural properties of C-1000, C-1000 Ar-600, C-1000 H_2-600 samples.

Samples	*R*	I_D/I_G	V_{total} /cm^3 g^{-1}	$D_{average}$ /nm	$S_{Langmuir}$ /cm^2 g^{-1}	S_{BET} /cm^2 g^{-1}
C-1000	4.12	1.10	0.1441	7.24	98.15	79.60
C-1000 Ar-600	4.20	1.11	0.0870	6.04	71.24	57.64
C-1000 H_2-600	3.39	1.16	0.0576	8.20	34.85	28.09

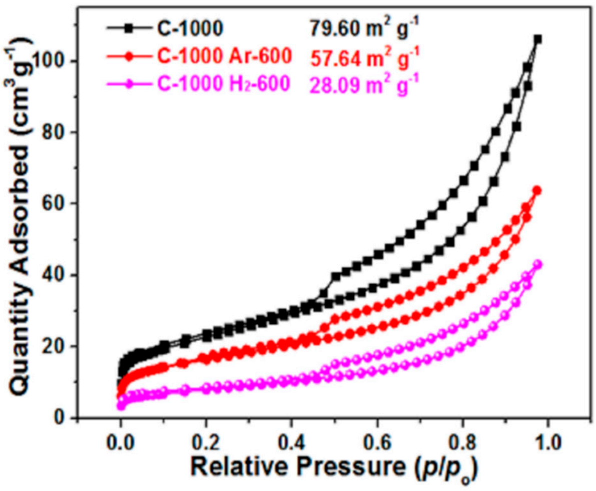

Figure S6. N_2 adsorption/desorption isotherms of C-1000, C-1000 Ar-600, C-1000 H_2-600 samples.

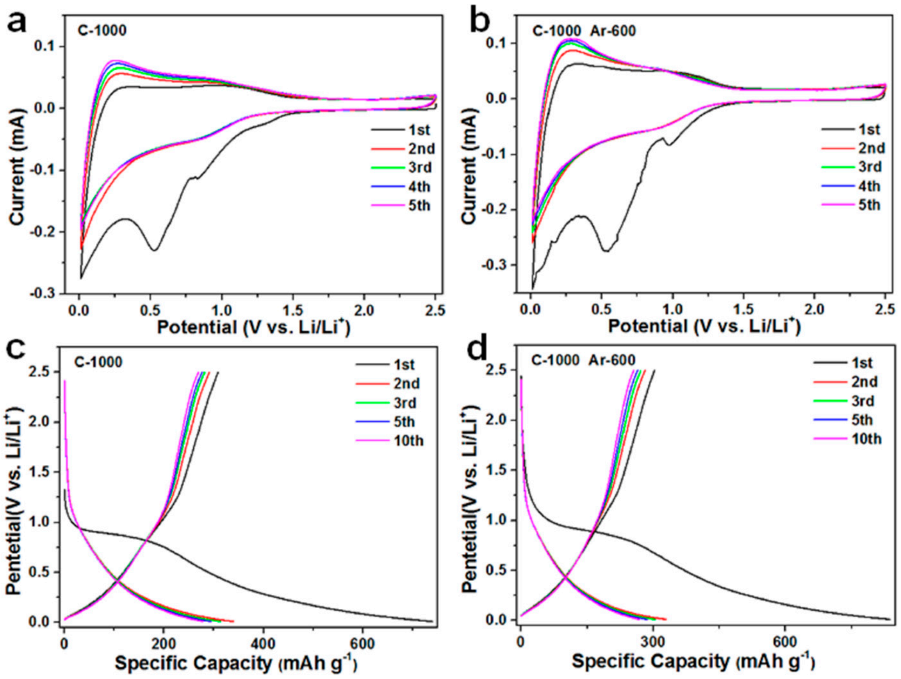

Figure S7. CV curves of (**a**) C-1000 and (**b**) C-1000 Ar-600 at 0.2 mV s^{-1} under the potential range of 0.01~2.5 V, Galvanostatic charge-discharge of (**c**) C-1000 and (**d**) C-1000 Ar-600 at 0.05 A g^{-1} in LIBs.

Table S6. Initial discharge/charge capacities and initial Coulombic efficiency of the three materials at 0.05 A g^{-1}.

Samples	Discharge capacity (mA h g^{-1})	Charge capacity (mA h g^{-1})	Initial coulombic efficiency (%)
C-1000 H_2-600	1135.8	416.4	35.85
C-1000 Ar-600	739.8	308.5	41.70
C-1000	836.0	302.6	36.17

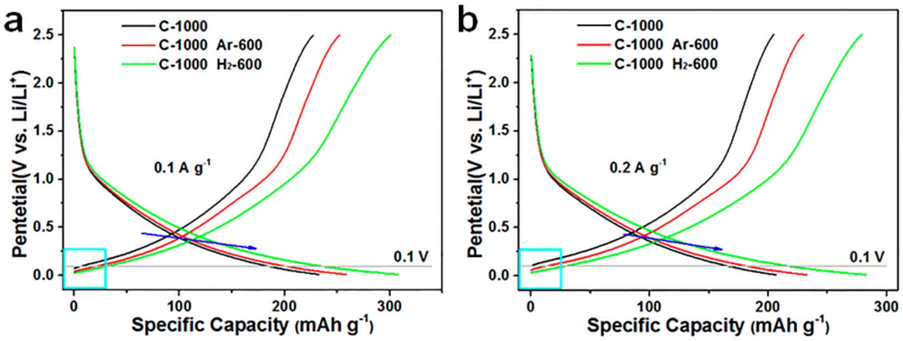

Figure S8. Galvanostatic charge-discharge of C-1000, C-1000 Ar-600 and C-1000 H_2-600 at (**a**) 0.1 A g^{-1} and (**b**) 0.2 A g^{-1} in LIBs.

Table S7. The discharge capacities (from the fifth cycle at the corresponding current density) of the three materials at different current densities in LIBs (the results of the commercial graphite are gained in our group).

Current density (A g^{-1})	C-1000 (mA h g^{-1})	C-1000 Ar-600 (mA h g^{-1})	C-1000 H_2-600 (mA h g^{-1})	Graphite (mA h g^{-1})
0.05	286.17	294.97	383.52	----
0.1	232.48	258.65	307.70	341.9
0.2	206.33	232.81	282.48	303.9
0.5	176.68	205.32	248.32	252.1
1.0	161.13	182.03	**223.98**	**198.5**
2.0	145.34	161.42	**196.97**	**109.1**
5.0	----	----	----	42.8
0.1	259.90	275.52	311.44	327.7
0.05	277.93	299.03	357.45	----

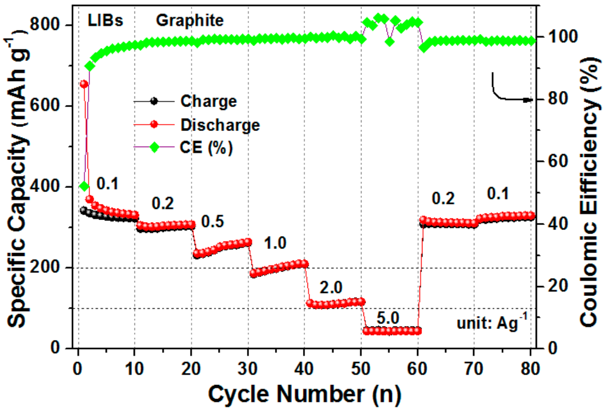

Figure S9. The rate performance of graphite.

Table S8. Comparison of C-1000 H_2-600 versus the recently reported coal-based carbonaceous anodes for LIBs.

**Materials**	**Rate capacity (mA h g^{-1})**	**Capacity retention (mA h g^{-1})**
Coal into a nonporous graphite[1]	104 at 1.0 A g^{-1}, 65 at 2.0 A g^{-1}	167 at 0.5 A g^{-1} after 1000 cycles
Carbon-coated sp^2 graphite[2]	115.3 at 2.0 A g^{-1}	--------
Coal-derived synthetic graphite[3]	160 at 1.0 A g^{-1}	--------
Synthetic graphite from bituminous coal, BCG-2800[4]	182 at 0.75 A g^{-1}, 134.8 at 2.0 A g^{-1}	--------
Coal-Derived Few-Layer Graphene, S5% Fe-Ni sample[5]	131.14 at 0.75 A g^{-1}, 80.37 at 2.0 A g^{-1}	177.78 at 0.372 A g^{-1} after 100 cycles
Pitch-based carbon microbeads, PCB2603-1000[6]	155.0 at 1.0 A g^{-1}, 134 at 2.0 A g^{-1}	250 at 0.5 A g^{-1} after 200 cycles
Pitch-Based Laminated Carbon[7]	220.0 at 1.0 A g^{-1}, 175 at 2.0 A g^{-1}	200 at 0.3 A g^{-1} after 200 cycles
MCMBs from D-SO[8]	230.0 at 1.0 A g^{-1}, 195 at 2.0 A g^{-1}	280 at 0.5 A g^{-1} after 400 cycles
Dry Quenched Coke, C-1000 H_2-600 (**This work**)	223.98 at 1.0 A g^{-1}, 196.97 at 2.0 A g^{-1}	205.53 at 2.0 A g^{-1} after 1000 cycles

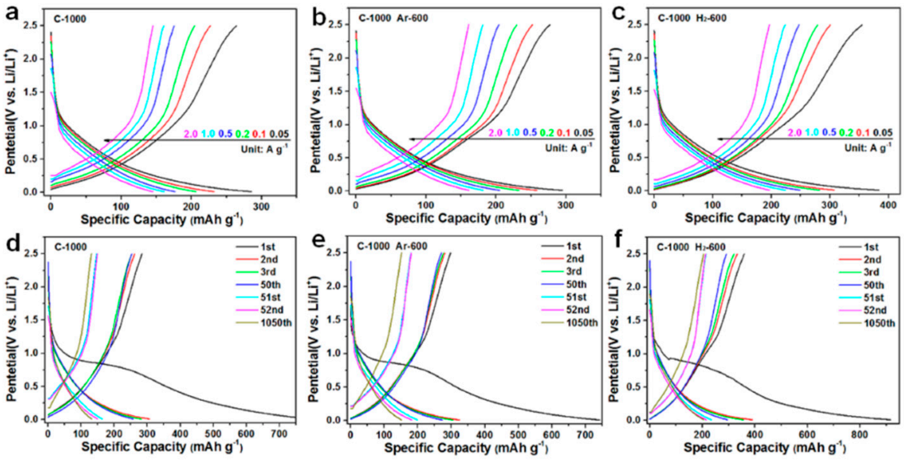

Figure S10. Galvanostatic charge-discharge of (**a**,**d**) C-1000, (**b**,**e**) C-1000 Ar-600 and (**c**,**f**) C-1000 H_2-600 at different current densities in LIBs.

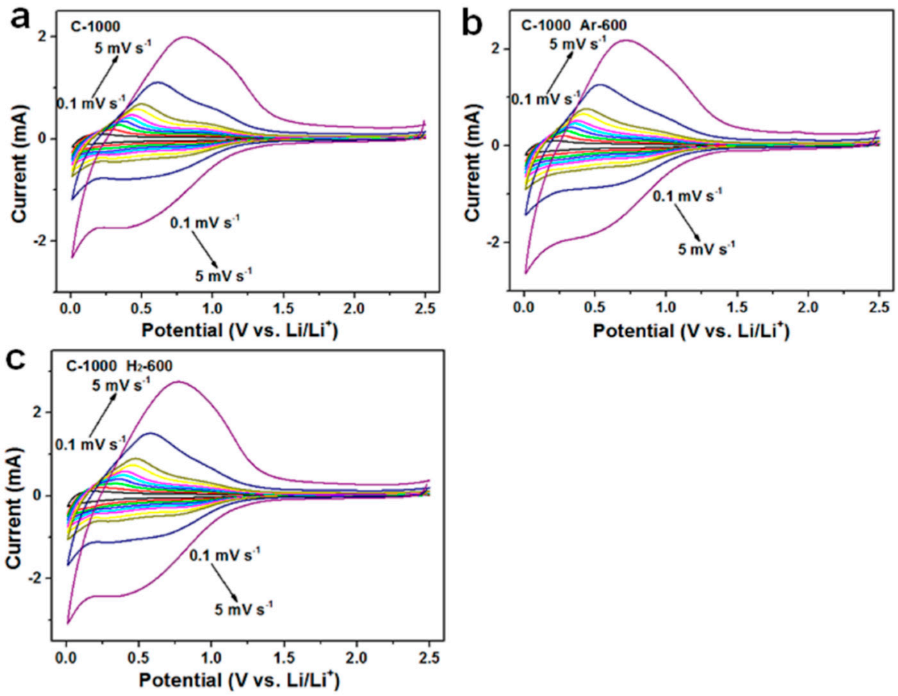

Figure S11. CV curves of (**a**) C-1000, (**b**) C-1000 Ar-600 and (**c**) C-1000 H_2-600 in LIBs at various scan rates under the potential range of 0.01~2.5 V.

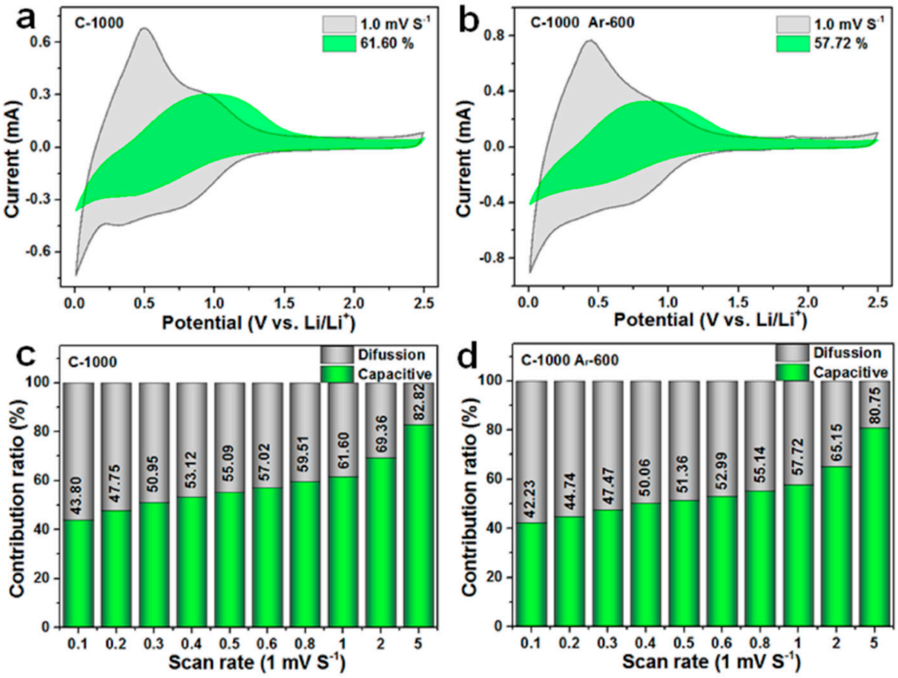

Figure S12. Capacitive contribution ratios (green) of the capacitive process for (**a**) C-1000, (**b**) C-1000 Ar-600 at a scan rate of 1 mV s^{-1}, Capacitive charge-storage contribution of (**c**) C-1000, (**d**) C-1000 Ar-600 at various scan rates from 0.1 to 5 mV s^{-1} in LIBs.

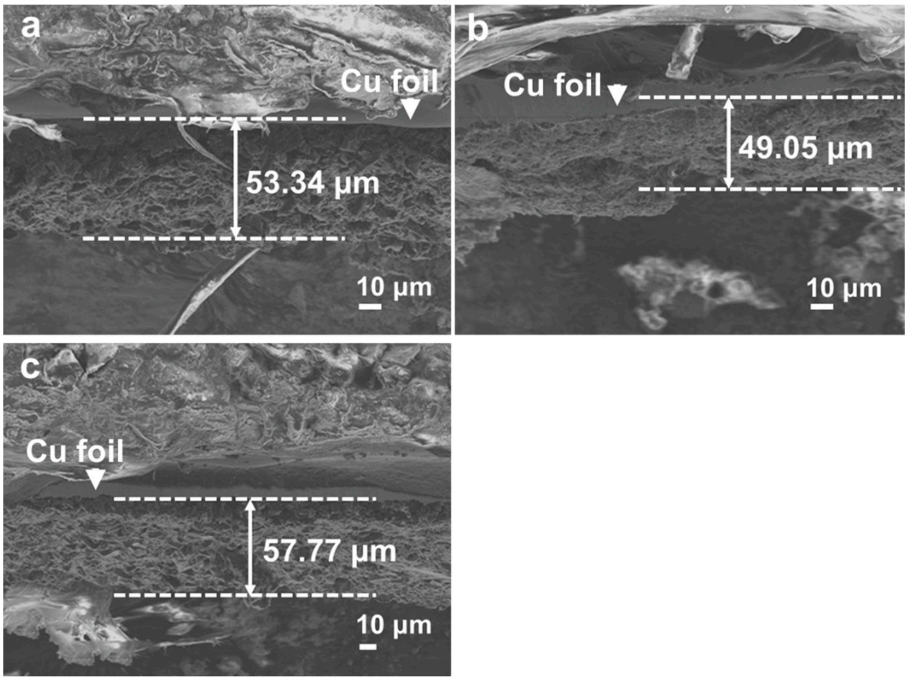

Figure S13. SEM images of the electrode thickness of (**a**) C-1000, (**b**) C-1000 Ar-600 and (**c**) C-1000 H_2-600 on Cu foil.

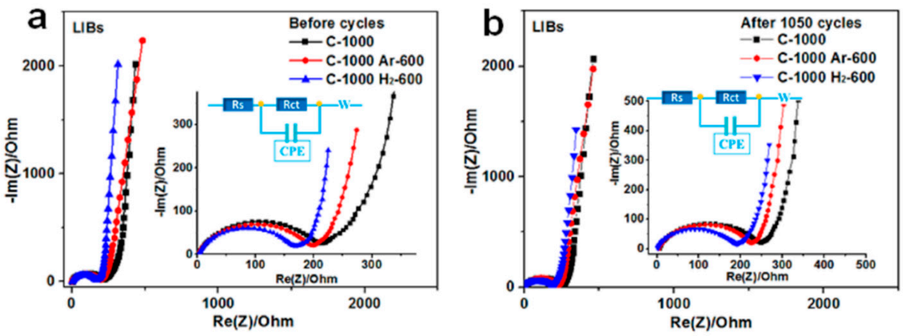

Figure S14. Electrochemical impedance spectra of C-1000, C-1000 Ar-600, C-1000 H_2-600 (**a**) before cycling, and (**b**) after 1050 cycles.

Table S9. Electrochemical impedance spectra of C-1000, C-1000 Ar-600, C-1000 H_2-600 before cycling and after cycling (from the 1050 cycles).

**Samples**	**Before cycling**			**After cycling**		
	**R_s (Ohm)**	**R_{ct} (Ohm)**	**CPE**	**R_s (Ohm)**	**R_{ct} (Ohm)**	**CPE**
C-1000 H_2-600	2.542	164.2	0.82	1.811	179.4	0.85
C-1000 Ar-600	1.987	188.6	0.82	3.902	220.0	0.83
C-1000	2.227	199.2	0.84	3.776	235.0	0.84

Table S10. The element contents from XPS of the C-1000 H_2-600 samples at different stages of discharge to 1.20 V and 0.20 V, charge to 0.50 V and 2.2 V states in LIBs.

**Elements**	**Percentage (at %)**			
	**Discharge 1.2 V**	**Discharge 0.2 V**	**Charge 0.5 V**	**Charge 2.2 V**
Li	30.18	30.93	28.60	23.21
F	21.62	34.95	22.00	19.83
C	28.15	22.28	18.13	20.33
O	18.87	10.97	30.38	34.64
P	1.18	0.87	0.89	1.98

Table S11. Carbon bonding analysis of the C-1000 H_2-600 samples at different stages of discharge to 1.20 V and 0.20 V, charge to 0.50 V and 2.2 V states in LIBs.

**Carbon Bonding**	**Percentage (at %)**			
	**Discharge 1.2 V**	**Discharge 0.2 V**	**Charge 0.5 V**	**Charge 2.2 V**
Li_2C_2	0.62	9.94	0.22	0.00
C-C	49.25	41.69	55.05	52.07
Defects	10.18	3.04	1.94	9.77
C-OH	15.91	19.75	22.07	18.56
C=O	3.82	2.73	0.40	0.37
COOH	9.09	7.81	10.89	10.15

π-π^*	11.13	15.04	9.43	9.08

Table S12. Oxygen bonding analysis of the C-1000 H_2-600 samples at different stages of discharge to 1.20 V and 0.20 V, charge to 0.50 V and 2.2 V states in LIBs.

**Oxygen Bonding**	**Percentage (at%)**			
	**Discharge 1.2 V**	**Discharge 0.2 V**	**Charge 0.5 V**	**Charge 2.2 V**
C=O	68.11	62.69	38.82	22.10
C-OH	17.91	28.63	43.72	40.01
COOH	13.98	8.67	17.46	37.89

Table S13. Lithium bonding analysis of the C-1000 H_2-600 samples at different stages of discharge to 1.20 V and 0.20 V, charge to 0.50 V and 2.2 V in LIBs.

**Lithium Bonding**	**Percentage (at%)**			
	**Discharge 1.2 V**	**Discharge 0.2 V**	**Charge 0.5 V**	**Charge 2.2 V**
Li_2O	0.00	0.63	1.14	7.98
COOLi/C-OLi	21.94	43.02	38.29	17.04
Li_2CO_3	59.55	50.23	46.67	35.26
LiF	18.51	6.12	13.90	39.72

References

1. Han, L.; Zhu, X.; Yang, F.; Liu, Q.; Jia, X. L. Eco-conversion of Coal into a Nonporous Graphite for High-performance Anodes of Lithium-ion Batteries. *Powder Technol*. **2021**, 382, 40-47.
2. Ma, Z.; Zhuang, Y. C.; Deng, Y.M.; Song, X.N.; Zuo, X.X.; Xiao, X.; Nan, J.M. From Spent Graphite to Amorphous sp(2) + sp(3) Carbon-coated sp(2) Graphite for High-performance Lithium Ion Batteries. *J. Power Sources* **2018**, 376, 91-99.
3. Shi, M.; Song, C.L.; Tai, Z.G.; Zou, K.Y.; Duan, Y.; Dai, X.; Sun, J.J.; Chen, Y.Z.; Liu, Y.N. Coal-derived Synthetic Graphite with High Specific Capacity and Excellent Cyclic Stability as Anode Material for Lithium-ion Batteries. *Fuel* **2021**, 292, 120250.
4. Xing, B.-L.; Zhang, C.T.; Cao, Y.J.; Huang, G.X.; Liu, Q.R.; Zhang, C.X.; Chen, Z.F.; Yi, G.Y.; Chen, L.J.; Yu, J.L. Preparation of Synthetic Graphite from Bituminous Coal as Anode Materials for High Performance Lithium-ion Batteries. *Fuel Process Technol* **2018**, 172, 162-171.
5. Islam, F.; Wang, J.L.; Tahmasebi, A.; Wang, R.; Moghtaderi, B.; Yu, J.L. Microwave-Assisted Coal-Derived Few-Layer Graphene as an Anode Material for Lithium-Ion Batteries. *Materials* **2021**, 14, 6468.
6. Yuan, M.; Cao, B.; Meng, C.Y.; Zuo, H.M.; Li A.; Ma, Z.K.; Chen, X.H.; Song, H.-H. Preparation of Pitch-based Carbon Microbeads by a Simultaneous Spheroidization and Stabilization Process for Lithium-ion Batteries. *Chem. Eng. J.* **2020**, 400, 125948.
7. Yang, T.; Song, Y.; Tian, X.D.; Song, H.-H.; Liu, Z.J. Pitch-Based Laminated Carbon Formed by Pressure Driving at Low Temperature as High-Capacity Anodes for Lithium Energy Storage Systems. *Chem. Eur. J.* **2020**, 26, 16514-16520.
8. Guo, A.J.; Wang, F.; Jiao, S.H.; Ibrahim, U.K.; Liu, D.; Liu, H.; Chen, K.; Wang, Z.-X. Preparation of Mesocarbon Microbeads as Anode Material for Lithium-ion Battery by Thermal Polymerization of a Distillate Fraction from an FCC Slurry Oil after Hydrofining with Suspended Catalyst. *Fuel* **2020**, 276, 118037.
